# Comparative Transcriptomics Reveals Clues for Differences in Pathogenicity between *Hysterothylacium aduncum*, *Anisakis* *simplex* sensu stricto and *Anisakis pegreffii*

**DOI:** 10.3390/genes11030321

**Published:** 2020-03-18

**Authors:** Serena Cavallero, Fabrizio Lombardo, Marco Salvemini, Antonella Pizzarelli, Cinzia Cantacessi, Stefano D’Amelio

**Affiliations:** 1 Department of Public Health and Infectious Diseases, Sapienza University of Rome, Piazzale Aldo Moro 5, 00185 Rome, Italy; serena.cavallero@uniroma1.it (S.C.); fabrizio.lombardo@uniroma1.it (F.L.); antonella.pizzarelli@uniroma1.it (A.P.); 2 Department of Biology, University of Naples Federico II, Corso Umberto I, 40, 80138 Naples, Italy; marco.salvemini@unina.it; 3 Department of Veterinary Medicine, University of Cambridge, Madingley Road, Cambridge CB3 0ES, UK; cinziacantacessi@gmail.com

**Keywords:** *Hysterothylacium*, *Anisakis*, transcriptome, pharyngeal transcripts, DEGs

## Abstract

Ascaridoid nematodes are widespread in marine fishes. Despite their major socioeconomic importance, mechanisms associated to the fish-borne zoonotic disease anisakiasis are still obscure. RNA-Seq and de-novo assembly were herein applied to RNA extracted from larvae and dissected pharynx of *Hysterothylacium aduncum* (HA), a non-pathogenic nematode. Assembled transcripts in HA were annotated and compared to the transcriptomes of the zoonotic species *Anisakis simplex* sensu stricto (AS) and *Anisakis pegreffii* (AP). Approximately 60,000,000 single-end reads were generated for HA, AS and AP. Transcripts in HA encoded for 30,254 putative peptides while AS and AP encoded for 20,574 and 20,840 putative peptides, respectively. Differential gene expression analyses yielded 471, 612 and 526 transcripts up regulated in the pharynx of HA, AS and AP. The transcriptomes of larvae and pharynx of HA were enriched in transcripts encoding collagen, peptidases, ribosomal proteins and in heat-shock motifs. Transcripts encoding proteolytic enzymes, anesthetics, inhibitors of primary hemostasis and virulence factors, anticoagulants and immunomodulatory peptides were up-regulated in AS and AP pharynx. This study represents the first transcriptomic characterization of a marine parasitic nematode commonly recovered in fish and probably of negligible concern for public health.

## 1. Introduction

Nematodes belonging to the genera *Hysterothylacium* (Ascaridoidea: Raphidascarididae) and *Anisakis* (Ascaridoidea: Anisakidae) are heteroxenous parasites widely found in several marine aquatic organisms [1,2,3,4]. Their life cycles are indirect and complex, based on prey-predator relations. Adults of the genus *Hysterothylacium* are common parasites of predatory teleosts, whilst various species of marine fishes act as intermediate hosts by harboring larval stages that are infective to their definitive hosts [5]. Definitive and intermediate hosts of *Anisakis* are marine mammals and crustaceans, respectively, while fishes and squids can act as paratenic hosts [2]. The majority of the larvae are located in the visceral body cavity of infected fish; however, seldom larvae may migrate to the flesh fillets, sometimes before the death of the host [6].

Parasitic larvae in fishes intended for human consumption represent an economic and medical issue; indeed, not only their presence in edible portions causes economic losses on the processing chain and reduced attitude to consume [7] but, most importantly, the ingestion of living *Anisakis* larvae may cause a mild-to-severe disease known as anisakiasis. This fish-borne zoonosis is classified as gastric (GA), intestinal (IA) and extragastrointestinal anisakiasis depending on the localization of the larva(e) in the body of the infected human, and it may also cause sensitization to parasite allergens [8,9].

Within the Anisakidae, *Anisakis simplex* sensu lato (s.l.) and *Pseudoterranova decipiens* s.l are mainly responsible for anisakidosis in humans [10,11]. The pathogenic potential of *Hysterothylacium* spp. is still controversial; even if this species is widely distributed amongst numerous fish species, their involvement in human infection and pathology is questionable [12,13]. Indeed, in spite of knowledge of antigens shared between *Hysterothylacium* spp. and *A. simplex* s.l. [14], according to the European Food Safety Authority, the latter is the only parasite in fishery products implicated in human sensitization and allergy [15]. Recently, high throughput transcriptomics has been applied to investigations of the potential mechanisms of pathogenicity of *Anisakis* larvae, with particular emphasis on molecules with potential roles in parasitic migrating through tissues and allergen sensitization [16,17,18,19]; nevertheless, the pathogenic potential of several marine parasitic nematodes other than *Anisakis* is still underinvestigated.

Therefore, in the present study, we carried out an in-depth analyses of the whole repertoire of transcripts differentially expressed between the whole larvae and the pharyngeal tissues of the non-pathogenic marine parasite *Hysterothylacium aduncum* (HA). Moreover, the comparison to phylogenetically related pathogenic *Anisakis simplex* sensu stricto (AS’) and *Anisakis pegreffii* (AP) was carried out, aimed to identify and characterize molecules involved in mechanisms of pathogenicity and host-parasite interactions and to evaluate allergenic potential of HA molecules.

## 2. Materials and Methods

### 2.1. Parasite Samples

Between 2015 and 2017, specimens of the Atlantic mackerel *Scomber japonicus*, and the European anchovy *Engraulis encrasicolus* and the European pilchard *Sardina pilchardus* were collected from the FAO 27 (North-East Atlantic region) and the FAO 37 fishing areas (Mediterranean basin), respectively, and dissected for the detection of anisakid larvae. In particular, viscera and fillets of each fish were visually inspected under a stereomicroscope; parasites were collected, washed and stored in filtered sterile PBS for subsequent isolation of the pharyngeal tissues (PX) [17]. The remainder of the larva (WL) was separated and both PX and WL were stored for further nucleic acids extractions.

For isolation of DNA and total mRNA, samples were homogenized and processed using the TRIsure™ reagent (Bioline, London, UK), thus overcoming potential biases due to partial removal of larval tissues or organs. Genomic DNA, obtained as residue during RNA isolation, was used as template for molecular identification of parasite species, based on PCR-RFLP analysis of the nuclear ribosomal marker internal transcribed spacer (ITS) according to published diagnostic keys [20,21].

### 2.2. RNA Extraction, Quality Check, cDNA Library Construction and RNA-sequencing

Total RNA was extracted using the TRIsure™ reagent (Bioline London, UK) and contaminating genomic DNA was removed using the Turbo DNA-free kit (Life Technologies, Carlsbad, CA, USA). Three independent biological replicates were performed, each consisting of total RNA extracted from three WL and ten to fifteen PX of each AS, AP and HA, respectively (96 larvae in total). The quality of the extracted RNA was visually verified on agarose gel, while resulting yields were measured using the Take3 Module of Synergy™ HT Multi-Detection Microplate Reader (Biotek, Winooski, VT, USA) and a Bioanalyzer 2100 (Agilent Technologies, Santa Clara, CA, USA).

Strand-specific RNA libraries were produced using the NEBNext^®^ Ultra™ kit (New England Biolabs, Ipswich, MA, USA), according to manufacturers’ instructions. Then, ligated products of 150–400 bp were excised from agarose gels and PCR-amplified. Products were purified using a MinElute PCR purification kit (Qiagen, Hilden, Germany) and the quality of each cDNA library was verified on an Agilent 2100 Bioanalyzer.

For RNA-Seq, pooled single-end libraries were sequenced on a HiSeq2500 platform (Illumina, San Diego, CA, USA) using HiSeq Flow Cell v4 and 125 bp read length.

### 2.3. Bioinformatics Analyses of Sequence Data

Raw reads were trimmed and filtered for Illumina adaptor sequences, sequences with suboptimal read quality (i.e., PHRED score ≥ 25.0) and sequences shorter than 125 bp using Trimmomatic v0.36 [22]. A first attempt for assembly was made using the genome guided assembly approach, given the availability of “*Anisakis simplex*” transcriptome from the WormBase repository database at [23]. Moreover, after results of mapping showing low coverage and given the fragmented nature of the *Anisakis* genome available, clean reads of WL and PX of all taxa were pooled and used for a de-novo assembly using the Trinity software [24]. Trimmed filtered reads for each species were aligned to the corresponding de novo assembled transcriptomes using Bowtie, and the relative expression of each transcript was calculated using the RSEM package and the no filter, FPKM1 and FPKM10 parameters. The trimmed mean of M-values normalization method (TMM) was used for cross sample normalization, and the normalized expression of each transcript was presented as transcripts per million reads (TPM). Assembled transcripts with <10 aligned reads were eliminated from subsequent analyses [25].

Differentially expressed transcripts (DEGs) between WL and PX belonging to each AS, AP and HA were identified by edgeR package [26,27] in R (|logFC| > 2), using transcripts filtered according to the FPKM1 parameter. A false-discovery rate correction FDR < 0.05 was applied in order to control for errors associated with multiple testing [28].

Putative orthologue transcripts shared between all pairwise combinations among HA, AS and AP were identified using a Reciprocal Best Hit analysis on the longest Open Reading Frame (ORF), and by only retaining sequences aligned by a continuous region covering at least 30% of the query sequence (E-value cut-off: 1 × 10^−6^ and minimum hit coverage 30%) [29].

Transcript annotation were performed using Annocript [30], which combines the annotation of protein coding transcripts with the prediction of putative lncRNAs in whole transcriptomes. Lists of coding regions identified in each transcriptome (i.e., Coding Domains [CD]) were obtained using the BLASTn, tBLASTn and rpsBLAST algorithms. Gene Ontology (GO) classifications assigned to each transcript, according to the categories Biological Process (BP), Molecular Function (MF) and Cellular Component (CC), were extracted by querying the SwissProt, UniRef and TrEMBL databases.

The HA transcriptome was interrogated for the presence of putative homologues of allergens in the “WHO/IUIS (World Health Organization and International Union of Immunological Societies) registered *Anisakis* allergenic proteins list”, counting 14 allergens so far [31], to verify the presence of allergenic molecules also in this species.

### 2.4. Validation of Transcripts by Quantitative Real-Time PCR

To verify transcript abundance patterns from RNA-seq analysis, RT-qPCR reactions were performed on seven transcripts selected among those that were overexpressed in PX (two isoforms of Hsp90 for HA; two cysteine-rich protein and astacin for AP; cysteine-rich and ShK venom for AS). A reference transcript for each species (i.e., elongation factor beta subunit for *Anisakis* and ribosomal 40S subunit for *Hysterothylacium*) was included to accomplish the relative quantification. Three independent batches of WL and PX samples for each of the three species under study were obtained from *Scomber japonicus* and *Engraulis encrasicolus*, as described above.

Total RNA was extracted using Trisure^®^ reagent (Bioline) following manufacturer’s instruction and resuspended in DNAse-RNAse-free ultrapure ddH_2_O. Quantity and quality of RNA was evaluated by spectrophotometric measurement using the Take3 module of plate reader BioTek SynergyHT and GEN5™ software and agarose gel electrophoresis. DNAseI treatment (using Ambion DNA-free kit^®^, Life Technologies, Carlsbad, CA, USA) was followed by standard PCR to verify efficacy of treatment. DNAseI-treated total RNA (~1 μg for dissected tissues and 2–4 μg for whole larvae) was used as substrate to generate First-Strand cDNA using SuperScript II RT (Invitrogen) and OligodT (Invitrogen). The synthesized cDNA samples were diluted to 10 ng/μL: 2 μL of cDNA were used in each qPCR reaction. For each gene, a standard curve was included in each plate, together with those of endogenous reference genes (ribosomal 40S for HA, EFB for AS and AP).

Templates of cDNA for the standard curve were obtained from WL RNA samples, with five points of a 1:5 dilution series starting from 100 ng/reaction. cDNA templates were mixed with 2× PowerUp™ SYBR™ Green Master Mix (Applied Biosystem, Foster City, CA, USA) and specific primers as listed in S1 (Supplementary S1).

Reactions included an initial holding stage of 2 min at 50 °C and of 2 min at 95 °C, followed by 40 cycles of PCR (95 °C, 15 s; 60 °C, 1 min); a final stage for melting curves was included, with detection steps every 0.3 °C.

The relative amount of each transcript was obtained by the ratio between the amounts of target transcript and endogenous reference transcript calculated using their Ct values and the corresponding standard curves. The ratio was then compared between different samples, selecting a sample as calibrator.

The relative transcription levels normalized by reference gene (ng ratios) as obtained by RTqPCR analysis were therefore compared with abundance levels as detected by RNA-seq (FPKM, Fragments Per Kilobase Million), as values were used to obtain a Heat-Map. Values from replicate experiments were averaged. Finally, to obtain values suitable for statistical comparisons, we calculated (for each gene) a fold-change (FC) value as the ratio of abundance of each transcript over the group median. These values (plotted after conversion in log2 numbers) were used to evaluate the correlation between RNA-seq and qPCR methods, applying statistical evaluation throughout Spearman test.

## 3. Results

### 3.1. Samples and Species Identification

A total of 115 anisakid larvae were collected, i.e. 38 from Atlantic mackerels, 49 from European anchovies and 28 from European pilchards. Of these, 31 larvae collected from Atlantic mackerels were identified as AS, 37 larvae collected from European anchovies were identified as AP following restriction analysis with *Hinf*I [20]. Twelve larvae from European anchovies and all larvae from European pilchards were identified as HA [21]. Three WL and PX samples from each HA, AS and AP were subjected to high-throughput RNA-Seq as described above.

### 3.2. The Hysterothylacium aduncum, Anisakis simplex sensu stricto and A. pegreffii Transcriptomes

All material isolated and analyzed with Bioanalyzer showed RIN values higher than 8.4 (range 8.4–9.5). The number of raw reads obtained from each WL and PX samples from each AS, AP and HA is available from Table 1.

HA raw reads were submitted to SRA under the Bioproject number PRJNA601087, AS and AP raw reads under the Bioproject number PRJNA374530.

The draft genome sequence was available for *A. simplex* [23,32] and we assumed that was related to the species *A. simplex* sensu stricto. Raw reads generated for the three taxa HA, AS and AP were mapped to the existing genome assembly sequences [23,33]. The highest percentages of mapping were obtained for transcriptomes belonging to the same genus. Thus, approximately 90% of reads for AS-WL and AS-PX could be mapped to the available genome sequences, with 86% of these mapping only once. Percentage of mapping obtained for AP were similar to those showed by AS, while HA showed very low percentage of mapping (6% mapped; 3.5% mapped once). Summary of mapping results is available in S2 (Supplementary material S2).

As anticipated in the Materials and Methods section, given the fragmented status of the current AS draft genome sequence and due to the unavailability of a draft genome sequence specific for AP and HA, raw reads generated from WL and PX samples for the three species were assembled de novo. De-novo assembly of AS-WL and AS-PX raw reads (*n* = 70,465,563) yielded, following quality and expression filtering, 74,738 contigs (Table 1); pooled reads for AP-WL and AP-PX (59,779,475) yielded 77,406 contigs, while pooled reads for HA-WL and HA-PX (62,310,036) yielded 112,416 contigs.

A total of 1392 putative orthologues were shared between HA and AS; 2556 between HA and AP and 1536 between AS and AP. Complete list of putative orthologues is available in S3 (Supplementary material S3).

### 3.3. Differential Gene Expression Analyses of Hysterothylacium aduncum, A. simplex sensu stricto and A. pegreffii

**Figure 1:**
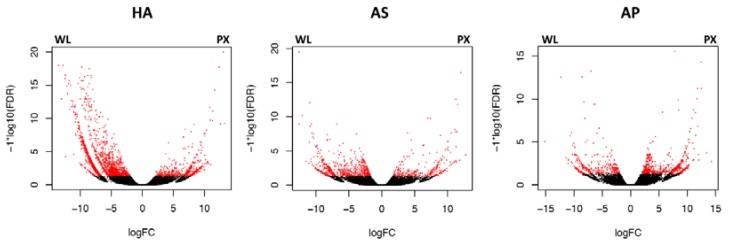
Tissue-specific gene expression. Volcano plots displaying the relative expression levels of transcripts upregulated in the pharynx (PX) compared with the whole larva (WL) in *Hysterothylacium aduncum* (HA), *Anisakis simplex* (s.s.) (AS) and *Anisakis pegreffii* (AP). The *x*-axis represents the log2 of the expression ratio for each transcript (tissue specific logCPM: whole body counts per million reads, logCPM, CPM); the *y*-axis represents the log10 of the *p*-value corrected for the false discovery rate. Red dots represent differentially expressed transcripts with logFDR < 0.05 (at least 2-fold difference in logCPM). Positive logFC values indicate transcripts enhanced in the pharynx subset (PX), while negative values indicate transcripts upregulated in the whole larva (WL).

Transcripts included in edgeR analyses testing the differentially expressed genes between WL and PX of each AS, AP and HA were 65,143 (44.9%), 38,531 (40.6%) and on 39,578 (30.1%) for HA, AS and AP, respectively (FDR < 0.05, Table 2; Figure 1).

For HA, a total number of 2288 differentially expressed genes were identified, of which 1817 were up regulated in WL and 471 in PX (Supplementary material S4).

Amongst WL upregulated transcripts, ~21% could be annotated (347 transcripts with PF and 175 unique PF) with the majority encoding collagens, von Willebrand factor type A, metallopeptidase (M14), lipases, SCAMP (secretory carrier membrane-trafficking proteins) and Patched proteins (transmembrane protein). Amongst the PX enriched transcripts, 172 (36.5%) could be annotated, including sequences encoding ribosomal proteins, collagens, recognition motifs for RNAs or carbohydrates metabolism and peptidases. Concerning, Gene Ontology analyses of conserved protein domains and biological processes, the presence of enriched transcripts on both WL and PX broadly related to stress and/or oxidative stress response were observed (Table 3). The remaining genes could not be annotated based on the information available in current public databases.

In AS, ~45% (128) of WL upregulated transcripts could be annotated, with the majority encoding peptidases (family M1 and S10), transmembrane proteins and transporters, and heath-shock proteins. Amongst the PX enriched transcripts, 256 (41.8%) were assigned pfam annotation; the most represented protein families were peptidases including zinc metalloproteinase, trehalase, carboxypeptidase and allergens as *Ancylostoma* secreted protein/Venom allergen.

In AP ~60% of transcripts upregulated in WL were annotated; the most represented protein families were linked to transporter (major facilitator superfamily transporter) and enzymatic activities (phospholipase B, peptidase family M1 domain, carboxylesterase family). For PX upregulated transcripts, ~40% could be annotated and the most represented protein families were linked to enzymatic activity of peptidases (zinc metalloproteinase nas-13) and others, such as lipase and Serine/threonine-protein kinase, allergens as Major allergen Ani s 1 and Ancylostoma secreted protein. The six most represented protein families identified in the transcriptomes of each AS, AP and HA are listed in Table 4.

**Figure 2:**
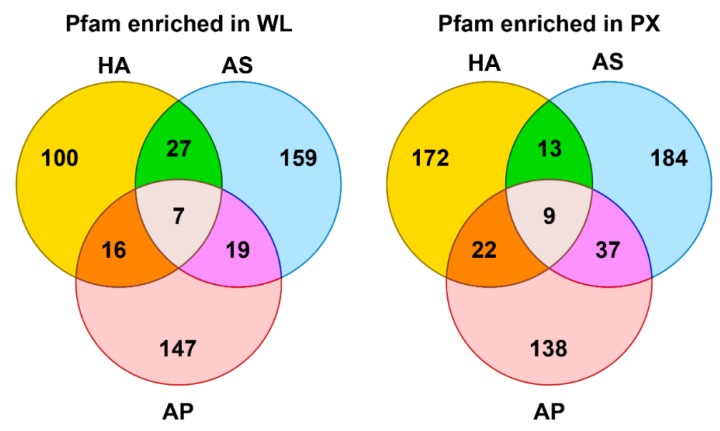
Venn diagrams. Venn diagrams showing numbers of p-families found enriched in the whole larvae (WL) and in the pharyngeal tissue (PX) of *Hysterothylacium aduncum* (HA), *Anisakis simplex* s.s. (AS) and *A. pegreffii* (AP).

Considering bioinformatics evidences here obtained on shared and unique observations for taxa under study (Figure 2, Venn diagram A and B), families and functions related to transporters (sugar, putative hormones, and superfamily membrane transport proteins), to enzymatic processes (mainly glycosyl hydrolases) and to pepsin inhibitors were shared by whole body larvae of all species. Members of peptidases (aspartic protease, zinc metalloproteinase) and other enzymatic processes (serine/threonine-protein kinase, putative aldehyde dehydrogenase family) were observed in pharyngeal regions of all species.

### 3.4. Transcripts Annotation

Annotation information linked to the transcriptomes of AS, AP and HA (WL and PX) were derived from the Annocript analyses.

Numbers of predicted coding regions of transcripts of each category of interest are reported in Table 5; because of the current unavailability of information on the transcriptome of HA, annotation details for this species are described below.

The repertoire of molecules identified in the HA transcriptome consisted of 21,784 predicted peptides inferred from 34,603 assembled transcripts; a total of 16,645 predicted peptides were inferred from 23,496 assembled transcripts in AS and 14,653 predicted peptides were inferred from 24,120 assembled transcripts in AP.

Annotation of pharyngeal enriched protein families in HA revealed a high presence of member of peptidases including astacin (peptidase family M12A pfam01400), of the stress-related heat shock protein HSP90 (pfam00183) and of cytochrome P450 (pfam00067). Protein families largely represented in the whole larva were the nematode cuticle collagen (pfam01484), collagen (pfam01391), the von Willebrand factor (pfam00092) and metallo-peptidases, in particular those belonging to the zinc carboxypeptidase group (pfam00246 M14). Biological processes largely represented in HA were related to protein folding, organismal development, with a great contribution of cuticle formation and response to stress (including oxidative stress). Among the 14 known *Anisakis* allergens, four were retrieved in HA transcriptome: the Kunitz serine protease inhibitor (Ani S1), the pan-allergens myosin and tropomyosin (Ani S2 and Ani S3, respectively) the recently described major allergen hemoglobin (Ani S 13).

Enriched molecules reported in pharyngeal tissue of AS were peptidases (family M12A pfam01400, family M13 pfam01431), aspartyl protease (pfam00026) and the CAP Cysteine-rich secretory protein family (pfam00188). Molecules enriched in AP pharyngeal tissue included peptidases (Astacin pfam01400; Lipase pfam01764; Peptidase_M13 pfam01431) and CAP cysteine-rich secretory proteins (pfam00188).

Both *Anisakis* species showed metallo-endopeptidase activity as the most frequent biological process associated to transcript up-regulated in the pharynx, whilst ribosomal and structural activities were the most represented BP terms associated to transcripts up-regulated in HA.

### 3.5. Relative Quantification of Validated Genes by Real-Time PCR

**Figure 3:**
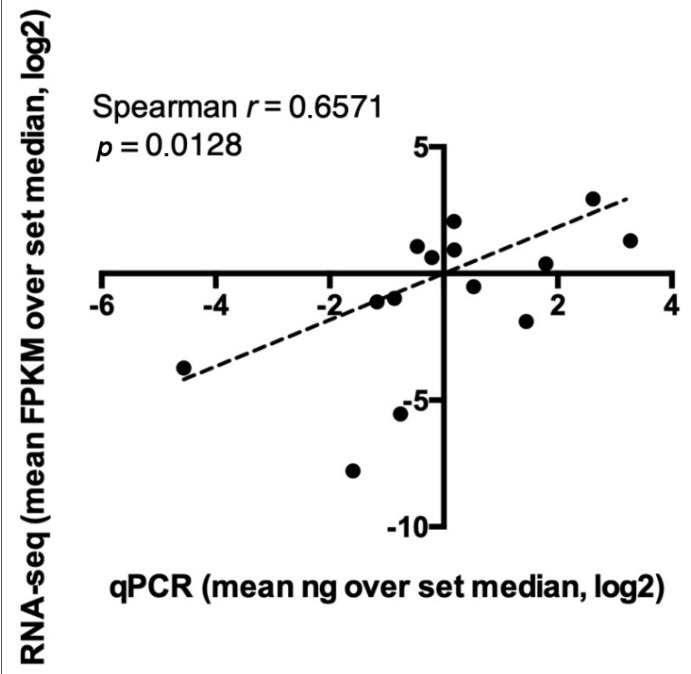
qPCR validation. Correlation between transcriptional abundance of 7 genes in both pharynx and whole larvae tissues revealed by qPCR and RNA-seq. Level of abundance is defined as the ratio between each sample value over the group median in both qPCR and RNA-seq approaches. To correlate datasets from the two techniques, statistical evaluation throughout Spearman test was performed and results are reported in the figure inset.

**Figure 4:**
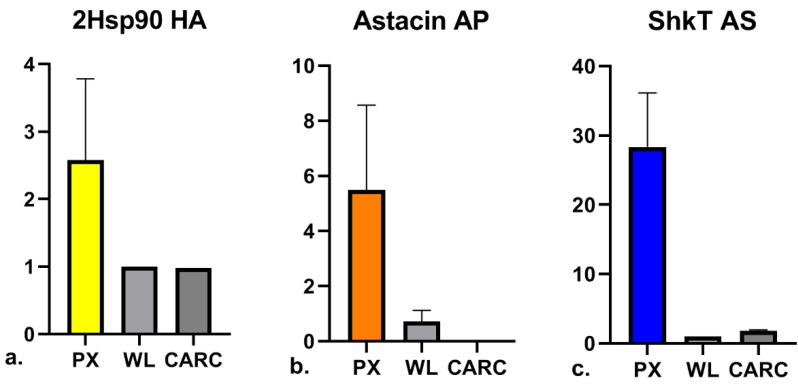
RTqPCR validation of transcript abundance. Graphs summarize the relative quantification obtained by Real Time PCR of transcripts selected among the PX enriched cluster in the three taxa. Representative bar plots, with standard errors indicated, are shown: (**a**). Hsp90 isoform 2 in HA; (**b**). astacin in AP; (**c**). Shk toxin in AS. Transcript abundance was investigated in dissected pharyngeal region (PX), in whole larvae (WL) and in the remaining carcasses from the dissections (CARC).

Transcriptional specificity and abundance of a few selected candidates were validated by quantitative Real Time PCR. Specific primers were designed for the amplification of 7 transcripts, listed in Table S1, that were selected according to the following criteria: (i) transcripts enrichment in pharyngeal samples compared to whole larvae and/or carcasses; (ii) transcripts absolute abundance in pharynxes (FPKM > 20 in all candidates, FPKM > 100 in 5/7 candidates) and (iii) involvement of the putative encoded peptides in functional pathways relevant for pathogenicity and/or host-parasite interactions. Results obtained from the relative quantifications by RT-qPCR of transcripts enriched in the pharyngeal body portion indeed confirmed the indications obtained from RNA-seq dataset in all species as shown in the Heat-Map (Table 6). The overall concordance between the abundance patterns revealed employing the two technical approaches is clear. Indeed, all the candidates analyzed showed increased transcript abundance in pharyngeal tissues compared to whole larvae with the only exception of *H. aduncum* 1Hsp90 where this evidence was not confirmed by RTqPCR. Moreover, correlation studies indicate statistically significant (*p* < 0.05) linear relationships between datasets obtained from the two methods for both pharyngeal and whole larvae samples analyzing data from the 7 selected candidates, as shown in the Figure 3. In particular, PX transcripts of the two Hsp90 isoforms in HA were found 1.5 to 3 times higher than in WL (Figure 4a). This trend was even more evident in the other species under study: cysteine-rich proteins and astacin transcripts in AP were 4.5 up to 8 times higher in the PX tissue compared to WL (Figure 4b), as well as in AS, PX transcripts of cysteine-rich protein were 1.5 times higher compared to WL transcripts and, finally, ShK toxin transcripts were up to 30 times higher in PX than WL (Figure 4c). List of primers and bar plots are available in S1.

## 4. Discussion

Anisakidosis is an emerging fish-borne zoonosis of major public health concern and despite the increasing concern on the impact of the disease, which is thought to be largely underestimated; our current understanding of the mechanisms of infection and clinical disease in humans is limited. Recently, some attempts have been made to clarify some aspect of infection using in-vivo tests with animal models [19] or to explore human immune responses using in-vitro systems [33], suggesting a general strong induction of immune reaction. However, studies on comparison of pathogenic anisakid species versus phylogenetically related non-pathogenic nematodes were thus far unavailable.

Here, we provide the first characterization of the entire set of molecules transcribed by the whole larva and pharyngeal tissue of *Hysterothylacium aduncum*, an ascaridoid nematode species related to anisakids, widely distributed in fishes.

The transcriptome of this parasite was here compared with those *A. pegreffii* and *A. simplex* sensu stricto, and to the *Anisakis* genome available in Wormbase, as a further attempt to corroborate the potential pathogenic role of specific molecules enriched in the pharyngeal tissues [17]

The de-novo assembly approach provided a similar number of transcripts for the three species and, after filtering for expression, the number of predicted genes was similar to those inferred from previous studies of *Anisakis* spp. and of other non-model species of parasitic nematodes [16,17,34]. As previously stated, a de-novo assembly approach was preferred to a genome-guided assembly based on the available draft genome sequence for *A. simplex.*

This may have provided an underestimation of orthologous genes. The number of full-length peptides predicted from de-novo assembled transcripts sequences were similar and congruent with filtered transcripts for all the three taxa of interest. However, the availability of a genomic resource only for *A. simplex* and the paucity of other comparative data led to a partial annotation with particular regard to enriched transcripts recovered in tissue of interest. Moreover, the higher number of shared pfam between AS and HA than between two phylogenetically close related taxa, such as AS and AP, may be the reflection of a fragmented and redundant transcriptome of HA, as suggested by the higher number of total trinity genes used for differential expression analyses.

The large recovery of peptidases proteins in all species may be related to the recruitment of such molecules in host-tissue invasion during larval migration, a typical behavior of the third-stage larvae of nematodes (a developmental form aimed to migrate into the host tissue and encapsulate until the availability of a suitable final host in which it matures into adult stage).

Larvae are transmitted along with a marine trophic web and, once ingested by fishes, they migrate digesting the extracellular matrix of gastrointestinal organs to reach the visceral cavity and/or to adhere to other organs such as liver and gonads. These enzymes may be responsible of pathogenic larval migratory behavior also in human cases of anisakiasis.

The possible reason for the scarce or negligible cases of human infections caused by *Hysterothylacium* may be related to the strong cellular response to stress (thermic and oxidative) that this species may produce. Devaney reviewed the role of thermoregulation in the life cycle of nematodes as a key factor for the ability to adapt to a variety of ecological niches, describing the regulation of HSF (Hsp70 and Hsp90) as extremely complex [35]. In fact, these genes can be activated by heat shock or other stress, as accumulation of denatured proteins or hormones-like proteins; in addition, many genes that are not classical Hsps may contain heat-shock elements (HSEs) in their upstream regions and may be responsive to heat shock as well. Moreover, a study on in-vitro cultivation of *H. aduncum* from third-stage larvae to egg-laying adults revealed that the best survival rates and percentage of moulting to L4 occurred at 13 °C, while at 37 °C nematodes survived only for few hours [4].

Given that the life cycle of *Hysterothylacium* occurs completely in benthic or pelagic crustaceans and teleosts (intermediate and definitive heterothermic hosts), while *Anisakis* become adult in homeothermic marine mammals, it can be supposed that high body temperatures, such as within human hosts, may not be a suitable niche for the survival of *Hysterothylacium*.

Peptidases were observed in all parasitic species, both at the whole body and pharyngeal level, but the recovery of only metallo-peptidases in *Hysterothylacium* sp., compared to the observation of several peptidases families in *Anisakis* spp., may suggest that these proteins could be related to invasive larval behavior, with relevant impact on the pathology in human infections, or to their ability to resist to the host’s body temperature. Moreover, allergens similar to AniS1 (i.e. Kunitz-like, pfam00014) were observed in *Hysterothylacium*, as well, but no venom-like proteins as those observed in *Anisakis* (i.e. *Ancylostoma* Toxin and Venom like, pfam 000188) were identified from whole larvae and pharyngeal enriched transcripts in *Hysterothylacium*.

Some of the recovered *Anisakis* pan-allergens in HA transcriptome have demonstrated strong cross-reactivity to homologous proteins in other invertebrates as crustaceans, shellfish and mites [36,37]. Although the hemoglobin Ani s 13 belongs to a conserved protein family, it seems not to be a cross-reacting antigen [38]. The apparent lack in HA of antigens specifically involved in IgE and IgG bound and trigger of allergic response in anisakiasis may be suggestive of limited ability of HA to induce a strong immune response.

Given the evidences obtained in the present and previous studies, the common fish parasitic nematode HA may probably represent a mere aesthetic problem related to consumers’ perception of food healthiness, rather than a menace to human health.

## Figures and Tables

**Table 1:** RNAseq results.

		HA	AS	AP	AS WB
	Total pooled reads	62,310,036	70,465,563	59,779,475	/
Assembly NO FILTER	Total trinity ‘genes’:	136,550	88,087	85,068	20,971
	Total trinity transcripts:	183,043	117,899	118,087	20,971
	Contig N50:	613	1236	1762	1197
Assembly FILTERED FPKM10	Total trinity ‘genes’:	30,254	20,574	20,840	9362
	Total trinity transcripts:	34,603	23,469	24,120	9362
	Contig N50:	341	839	1466	1218
Assembly FILTERED FPKM1	Total trinity ‘genes’:	112,416	74,738	77,406	15,811
	Total trinity transcripts:	145,023	94,906	101,018	15,811
	Contig N50:	504	1071	1521	1281

Data on the reads obtained from RNAseq of the three taxa under study (HA: *Hysterothylacium aduncum*; AS: *Anisakis simplex* sensu stricto; AP: *Anisakis pegreffii*; AS WB: data retrieved from Wormbase repository regarding “*Anisakis simplex*”).

**Table 2:** Results from the differential expression analyses.

		HA	AS	AP
Assembly filtered FPKM1	edgeR included transcripts	145,023	94,906	101,018
PX	*p* < 0.05	471	612	526
	*p* < 0.01	185	298	213
	Contig N50:	1178	2569	2366
WL	*p* < 0.05	1817	320	282
	*p* < 0.01	1107	122	127
	Contig N50:	993	2783	2298

DEGs performed on the whole larva (WL) vs. pharyngeal (PX) transcripts for *Hysterothylacium aduncum*, *Anisakis simplex* s.s. and *A. pegreffii*. Upregulated transcripts are indicated for WL and PX, with adjusted *p*-values and N50.

**Table 3:** Top five HA enriched transcripts. List of the five most enriched groups of transcripts in HA classified according to the conserved protein domain family and to the gene ontology (biological process and molecular function), with indication of total number of transcripts recovered, of enriched ones, and of *p*-value and adjusted *p*-value (FDR).

	ID	Total	UP	Description	*p*-Value	adjp
	**Pharyngeal enriched**					
Conserved domain Protein Family	pfam01400	63	8	Astacin (Peptidase family M12A)	3.34 × 10^−17^	1.76 × 10^−15^
	pfam00183	111	8	HSP90 protein	1.11 × 10^−9^	3.51 × 10^−8^
	pfam04379	3	3	DUF525 Protein of unknown function	3.85 × 10^−25^	6.09 × 10^−^^23^
	pfam01776	27	3	Ribosomal_L22e	2.57 × 10^−5^	5.08 × 10^−^^4^
	pfam00067	33	3	Cytochrome P450	1.8 × 10^−3^	2.59 × 10^−3^
	**Whole larva enriched**					
	pfam01484	126	38	Nematode cuticle collagen	3.70 × 10^−74^	8.55 × 10^−72^
	pfam01391	55	19	Collagen triple helix repeat	2.26 × 10^−41^	1.74 × 10^−39^
	pfam00092	41	16	VWA von Willebrand factor type A	2.18 × 10^−38^	1.26 × 10^−36^
	pfam00246	32	13	Peptidase_M14 Zinc carboxypeptidase	5.11 × 10^−32^	2.36 × 10^−30^
	pfam01674	18	12	Lipase_2	5.52 × 10^−42^	6.38 × 10^−40^
	**Pharyngeal enriched**					
Gene ontology Biological process	GO:0006412	758	20	translation	1.49 × 10^−6^	2.47 × 10^−5^
	GO:0006950	133	8	response to stress	2.72 × 10^−8^	6.30 × 10^−7^
	GO:0007067	72	4	mitosis	6.35 × 10^−4^	6,14 × 10^−3^
	GO:0007155	80	4	cell adhesion	1.54 × 10^−3^	1.27 × 10^−2^
	GO:0051301	85	4	cell division	2.47 × 10^−3^	1.91 × 10^−2^
	**Whole larva enriched**					
	GO:0006457	397	15	protein folding	2.84 × 10^−4^	1.30 × 10^−2^
	GO:0007275	82	11	multicellular organismal development	8.06 × 10^−16^	7.41 × 10^−14^
	GO:0006950	133	8	response to stress	8.85 × 10^−5^	5.42 × 10^−3^
	GO:0006979	75	5	response to oxidative stress	1.47 × 10^−4^	1.94 × 10^−3^
	GO:0007224	3	4	smoothened signaling pathway	1.89 × 10^−27^	3.47 x 10^−25^
	**Pharyngeal enriched**					
Gene ontology Molecular function	GO:0003735	767	20	structural constituent of ribosome	5.42 × 10^−8^	5.75 × 10^−6^
	GO:0004222	253	8	metalloendopeptidase activity	1.26 × 10^−5^	1.67 × 10^−4^
	GO:0005506	128	7	iron ion binding	1.09 × 10^−7^	5.80 × 10^−6^
	GO:0020037	192	6	heme binding	1.46 × 10^−3^	1.50 × 10^−2^
	GO:0016705	56	4	oxidoreductase activity, acting on paired donors	9.41 × 10^−6^	2.49 × 10^−5^
	**Whole larva enriched**					
	GO:0042302	238	65	structural constituent of cuticle	4.39 × 10^−149^	5.93 × 10^−147^
	GO:0003700	385	38	sequence-specific DNA binding transcription factor	3.88 × 10^−29^	1.31 × 10^−27^
	GO:0043565	338	37	sequence-specific DNA binding	2.76 × 10^−32^	1.86 × 10^−30^
	GO:0005524	3496	35	ATP binding	3.34 × 10^−5^	3.54 × 10^−3^
	GO:0016787	377	16	hydrolase activity	8.91 × 10^−5^	8.59 × 10^−3^

**Table 4:** Top six pfam (PF) from DEGs analyses. List of the six most represented protein families with codes descriptions resulted from differential expression analyses performed on the whole larva (WL) vs. pharyngeal (PX) transcripts for *Hysterothylacium aduncum*, *Anisakis simplex* s.s. and *A. pegreffii*. Shared PF are underlined.

		HA	Description	AS	Description	AP	Description
PX	*p* < 0.05	471		612		526	
	Annotated transcripts	172		184		224	
	Six most represented pfam	PF01484 PF00238 PF04758 PF04516 PF02738 PF02064	Collagen Ribosomal protein Ribosomal protein Transcription factor Dehydrogenase Import receptor	PF01400 PF01431 PF00188 PF01204 PF01663 PF01826	Astacin Peptidase M13 CAP Hydrolase Phosphodiesterase TIL domain	PF00188 PF01400 PF01431 PF01764 PF00014 PF01060	CAP superfamily Astacin Peptidase M13 Lipase Kunitz BPTI Transthyretin-like
WL	*p* < 0.05	1817		282		320	
	Annotated transcripts	347		128		187	
	Six most represented pfam	PF01484 PF00092 PF00246 PF01391 PF01674 PF04144	Cuticole collagen von Willebrand factor Peptidase M14 Collagen Lipase SCAMP family	PF01060 PF01433 PF07857 PF00450 PF00012 PF07690	Transthyretin-like Peptidase M1 Transmembrane Serine carboxypeptidase Hsp70 Major Facilitator	PF01433 PF00083 PF07690 PF00307 PF00135 PF00046	Peptidase M1 Sugar transporter Major facilitator EB1 motif Carboxylesterase Homeodomain

**Table 5:** Numbers of predicted peptides obtained from the assembled transcripts after annotation analyses with Annocript. (HA: *Hysterothylacium aduncum*; AS: *Anisakis simplex* sensu stricto; AP: *Anisakis pegreffii*; ALL: pooled transcripts; PX_UP: transcripts enriched in pharyngeal tissue; WL_UP transcripts enriched in whole larvae; CD: conserved domains; GOBP: gene ontology_biological process; GOCC: gene ontology_cellular component; GOMF: gene ontology_molecular function; PW: pathways associated to the transcripts L1-3: first, second and third level).

	HA			AS			AP		
	ALL	PX_UP	WL_UP	ALL	PX_UP	WL_UP	ALL	PX_UP	WL_UP
>CD	>21,784	>206	>485	>16,645	>268	>184	>14,653	>240	>154
>GOBP	>21,274	>194	>307	>15,715	>154	>109	>16,270	>224	>215
>GOCC	>22,924	>185	>417	>16,991	>198	>138	>21,925	>311	>252
>GOMF	>33,185	>263	>593	>23,141	>303	>158	>16,365	>300	>283
>PWL1	>2828	>19	>27	>1814	>10	>11	>1715	>21	>17
>PWL2	>2845	>19	>28	>1848	>10	>11	>1745	>20	>17
PWL3	1310	4	11	832	5	7	740	7	7

**Table 6:** Heat-Map. RNA-seq abundance values of selected transcripts are reported (FPKM) and compared to relative quantifications obtained by RTqPCR (ng). Transcripts putative encoded peptides, contigs ID and species are also indicated. Colors in the Heat-Map are assigned separately for each species according to percentiles criteria, with blue referring to lower values (10 percentile) and yellow to higher values (90 percentile).

			RNAseq (FPKM)		RT-qCR (ng)	
Species	Symbol		PX	WL	PX	WL
*Hysterothylacium aduncum*	TRINITY_DN358353_c0_g4_i7	2Hsp90	130.80	0.31	1.11	0.32
*Hysterothylacium aduncum*	TRINITY_DN32835_c0_g1_i4	1Hsp90	285.98	18.58	1.10	2.65
*Anisakis pegreffii*	TRINITY_DN21666_c0_g2_i1	CAP	531.89	106.92	5.97	0.84
*Anisakis pegreffii*	TRINITY_DN20560_c0_g2_i7	CRP	89.58	1.48	3.36	0.57
*Anisakis pegreffii*	TRINITY_DN29135_c4_g14_i1	Astacin	169.56	35.03	9.41	0.53
*Anisakis simplex*	ASIM_0001574901	CRS	143.95	31.65	0.71	0.43
*Anisakis simplex*	ASIM_0001787701	ShKT	48.19	5.17	1.40	0.04

## Supplementary Materials

The following are available online at https://www.mdpi.com/2073-4425/11/3/321/s1, Supplementary material S1: List of primers used for validation of transcripts obtained by the RNA-seq analyses, followed by boxplots of relative quantification of selected transcripts for Real-Time validation, with indication of standard errors. Y axis: mean of ratios of nanograms of PX enriched genes on nanograms of reference genes; W axis: bars of pharynx (PX), the carcass (CARC) and the whole larvae (WL). HA in green: *Hysterothylacium aduncum*; AS in blue: *Anisakis simplex* sensu stricto; AP in orange: *Anisakis pegreffii*. Supplementary material S2: Boxplot of genome mapping results with indication of mapping percentages: unique mapping is reported in white, ambiguous mapping in black and unmapped in grey. HA: *Hysterothylacium aduncum*; AS: *Anisakis simplex* sensu stricto; AP: *Anisakis pegreffii*. Pharynx (PX), whole larvae (WL). Supplementary material S3: Best Reciprocal Hits results reported in six different tabs for each pair of species analyzed (HA vs. AS; HA vs. AP; AS vs. AP). Columns indicate clone (trinity transcripts code), hit (result from the comparison), description of info about transcripts resulted as best hit, e-value, coverage and identity. Supplementary material S4: Results from differential gene expression analysis in HA. All EdgeR included transcripts are reported in the first tab, WL enriched transcripts are reported in the second tab and PX enriched transcripts in the third tab, with indication of logFC, logCPM, PValue and FDR.
