# Supplementary material for: Comparative Transcriptomics Reveals Clues for Differences in Pathogenicity between Hysterothylacium aduncum, Anisakis simplex sensu stricto and Anisakis pegreffii
Source: Genes (Basel). 2020 Mar 18;11(3):321. doi: 10.3390/genes11030321 (PMC7140869; doi:10.3390/genes11030321)
Supplement: Supplementary file 1 [file genes-11-00321-s001.zip › S1.pdf]

List of primers used for validation of transcripts obtained by the Rna-seq analyses.			
**Primer Name**	**Sequence**	**Parasitic species**	**Type of gene**
EFB/F	CCATGGGATGATACGGTGCA	AP and AS	Constitutive
EFB/R	ATGATACCGAACGCCACTGG	AP and AS	Constitutive
HA40SF	GTCGGTCGCTGTGATGGTTA	HA	Constitutive
HA40SR	ACATCGCCCCTTCATATGCA	HA	Constitutive
CRS/F	CTCGATTGGGATGTGCCCTT	AS	PX_upregulated
CRS/R	TTACCCGGTTGCTCGTATCG	AS	PX_upregulated
SHKF	AGGTGGTCAATTGCATCGGT	AS	PX_upregulated
SHKR	TGCCGGTTAGTTCAGCACAT	AS	PX_upregulated
CRP/F	TGGGTCGCCAACTTCGTAAA	AP	PX_upregulated
CRP/R	TGGGATGTGCCCTTAACACA	AP	PX_upregulated
AST_apF	TCCCTTTGGCCATAATGCGT	AP	PX_upregulated
AST_apR	GTCGTTGAAAGGTGCGTCAC	AP	PX_upregulated
CAPF	CCAGGCATTTTGATCCCGGA	AP	PX_upregulated
CAPR	CGATACGAGCAACCGGGTAA	AP	PX_upregulated
HA1Hsp90F	GGTGGCAGTTTCAAACCGTC	HA	PX_upregulated
HA1Hsp90R	GGCCTTCATGATACGCTCCA	HA	PX_upregulated
HA2Hsp90F	GAAGGTGGCTGTTTCGAACC	HA	PX_upregulated
HA2Hsp90R	TCCATGTTCGCTGACCATCC	HA	PX_upregulated

Boxplot of relative quantification of selected transcripts for Real-Time validation, with indication of standard errors. Y axis: mean of ratio of nanograms of PX enriched genes on ng of reference gene; W axis: bars of pharynx (PX), the carcass (CARC) and the whole larvae (WL). HA in green: *Hysterothylacium aduncum*; AS in blue: *Anisakis simplex* sensu stricto; AP in orange: *Anisakis pegreffii*.

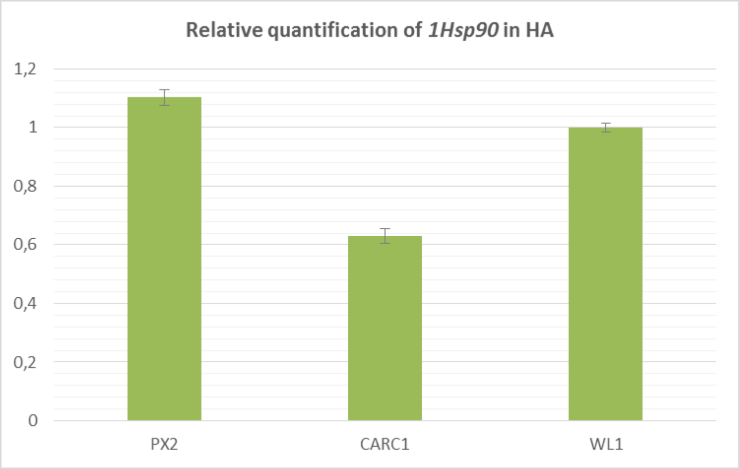

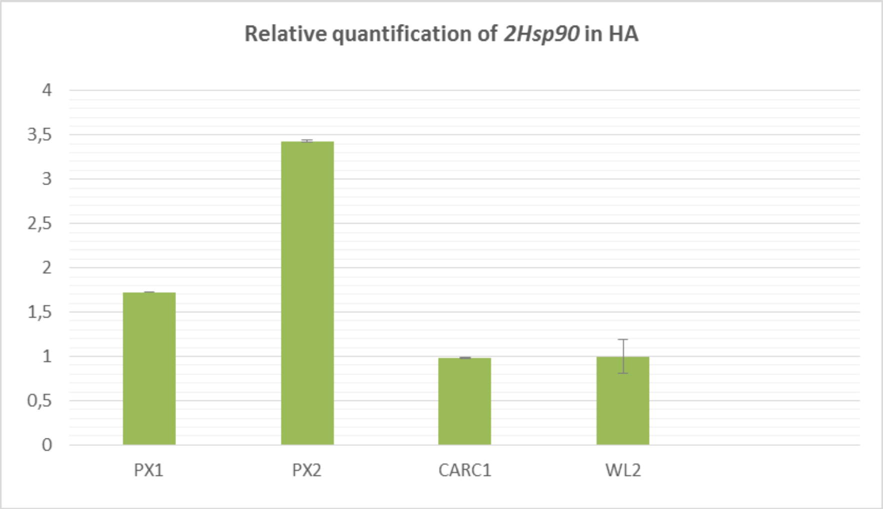
Relative quantification of *2Hsp90* in HA
4
3,5
3
2,5
2
1,5
1
0,5
0
PX1
PX2
CARC1
WL2

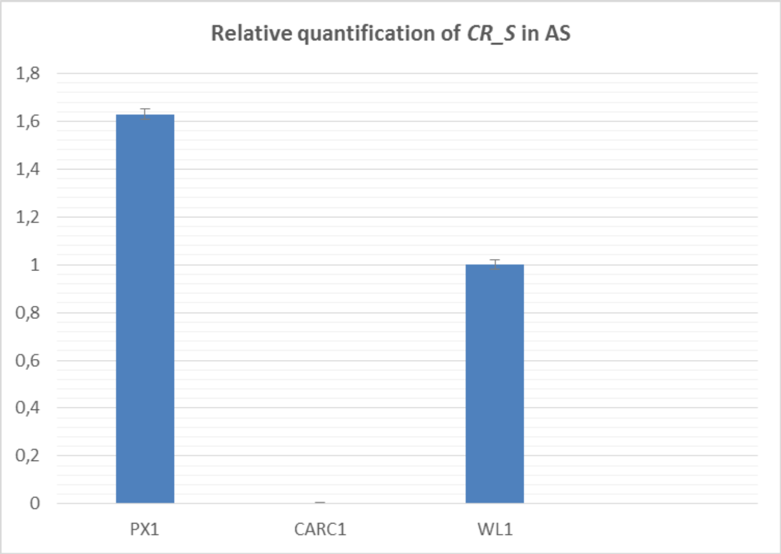
Relative quantification of *CR_S* in AS
1,8
1,6
1,4
1,2
1
0,8
0,6
0,4
0,2
0
PX1
CARC1
WL1

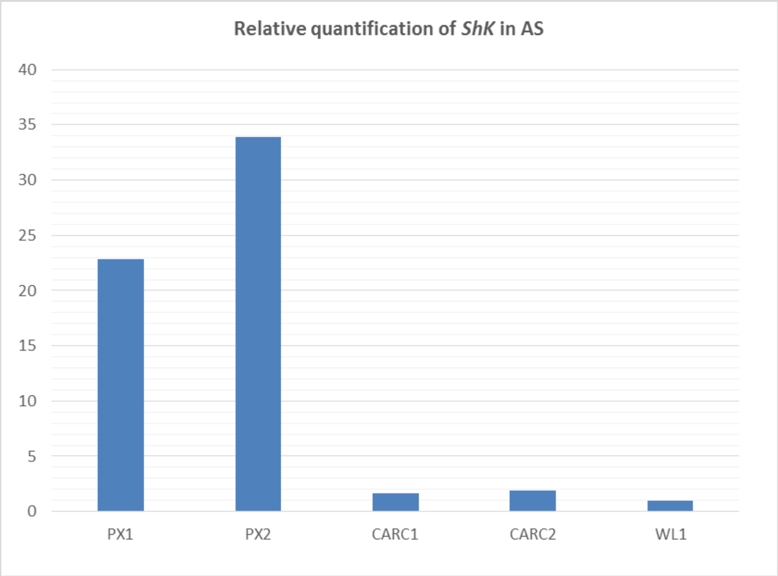
Relative quantification of *ShK* in AS
40
35
30
25
20
15
10
5
0
PX1
PX2
CARC1
CARC2
WL1

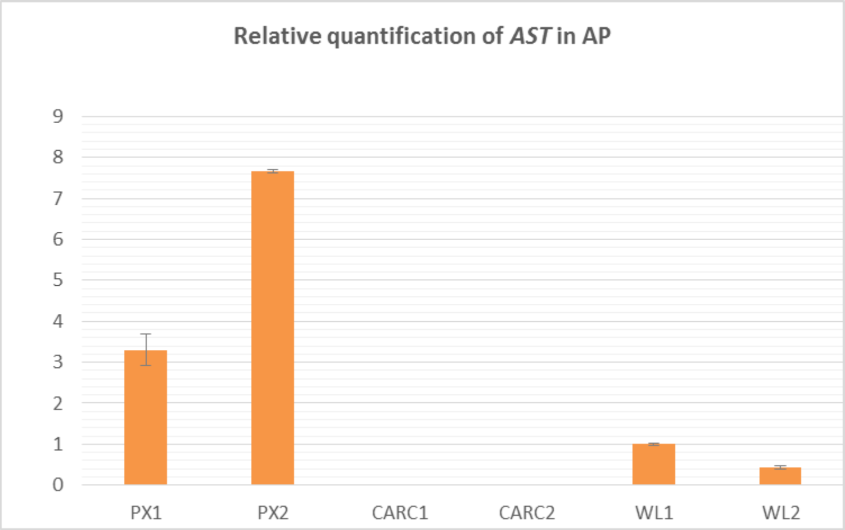
Relative quantification of AST in AP
9
8
7
6
5
4
3
2
1
0
PX1
PX2
CARC1
CARC2
WL1
WL2

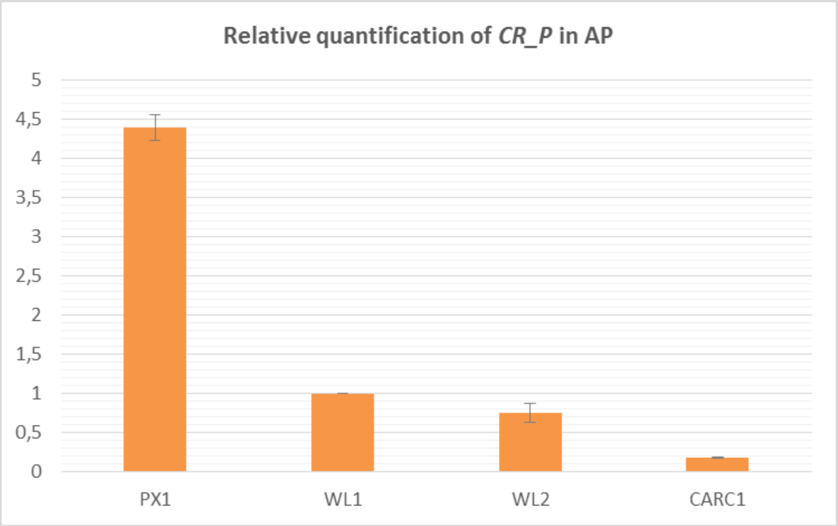
Relative quantification of CR_P in AP
5
4,5
4
3,5
3
2,5
2
1,5
1
0,5
0
PX1
WL1
WL2
CARC1

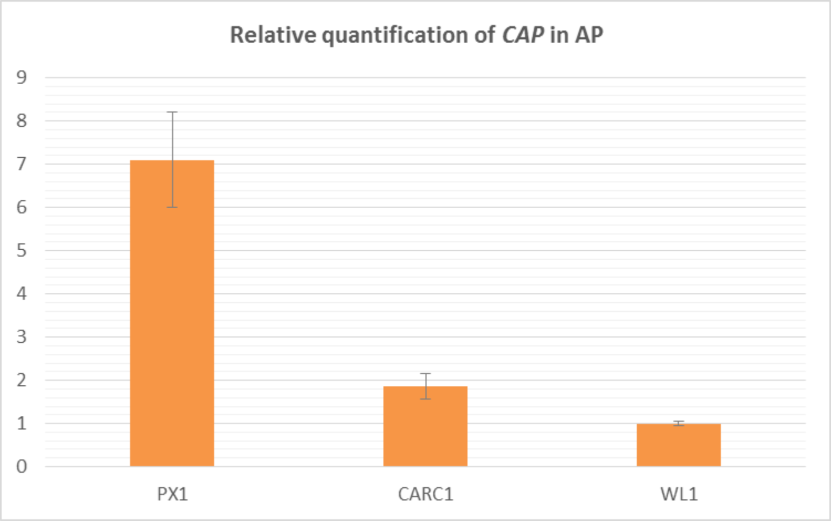
Relative quantification of CAP in AP
9
8
7
6
5
4
3
2
1
0
PX1
CARC1
WL1
