# Supplementary figures and images for: Comparative Transcriptomics Reveals Clues for Differences in Pathogenicity between Hysterothylacium aduncum, Anisakis simplex sensu stricto and Anisakis pegreffii
Source: Genes (Basel). 2020 Mar 18;11(3):321. doi: 10.3390/genes11030321 (PMC7140869; doi:10.3390/genes11030321)

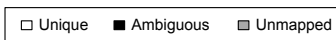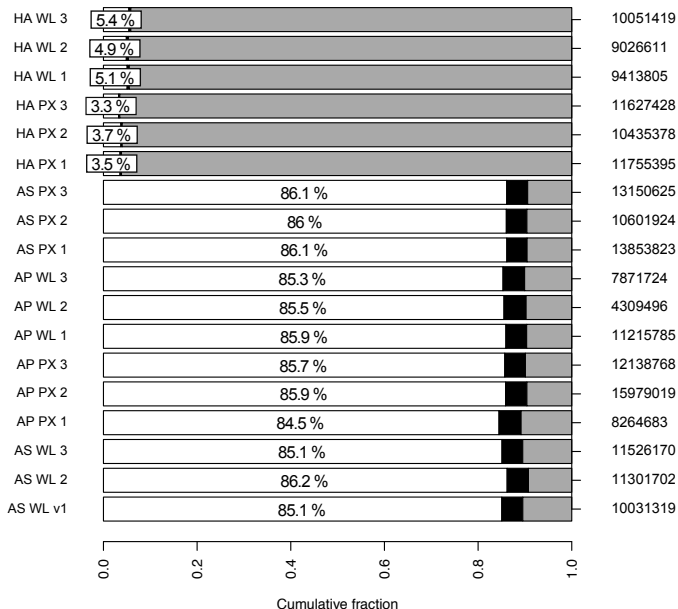

Supplement: Supplementary file 1 [file genes-11-00321-s001.zip › S2.pdf]
